# Peer-mentoring junior surgical trainees in the United Kingdom: a pilot program

**DOI:** 10.3402/meo.v18i0.20825

**Published:** 2013-04-18

**Authors:** Paul Vulliamy, Islam Junaid

**Affiliations:** 1 Department of General Surgery, Queen's Hospital, Romford, United Kingdom; 2 The Royal London Hospital, Whitechapel, London, United Kingdom

**Keywords:** mentoring, peer-mentoring, surgery, postgraduate training

## Abstract

**Background:**

Peer-mentoring has attracted substantial interest in various healthcare professions, but has not been formally integrated into postgraduate surgical training. This study aimed to assess the feasibility and acceptability of a peer-mentor scheme among junior surgical trainees in the United Kingdom.

**Method:**

Trainees entering the first year of core surgical training (CST) in a single postgraduate school of surgery were allocated a mentor in the second year of CST. Allocation was based on location of the initial clinical placement. An anonymised questionnaire regarding the mentorship scheme was sent to all participants in the third month following its introduction.

**Results:**

18 trainees participated in the scheme, of whom 12 (67%) responded to the questionnaire. All respondents had made contact with their allocated mentor or mentee, and no trainees had opted out of the scheme. Areas in which the mentees received guidance included examinations (83%), CV development (67%), and workplace-based assessments (67%). All respondents felt that the mentor scheme was a good addition to CST. Suggestions for improvement of the scheme included introduction of structured meetings and greater engagement with allocated mentors.

**Conclusions:**

A pilot peer-mentoring scheme was well received by junior surgical trainees. Consideration should be given to expansion of this scheme and more rigorous assessment of its value.

## Background

Junior doctors entering surgical training face a number of challenges, and some reports suggest that the dropout rate for surgical trainees in the early stages of training is higher than in other disciplines (1). In the United Kingdom and elsewhere, the introduction of the European Working-Time Directive (EWTD) places additional demands on trainees in surgery, with a greater pressure to attain competence within a constrained time period (2, 3). Postgraduate training institutions require the achievement of a myriad of targets during the course of training to demonstrate competency. In addition, the age-old challenges of completing membership examinations while developing a varied academic portfolio can constitute further stressors, particularly in the context of ever-more competitive applications for higher specialty training (4).

Mentorship has long been an important component of training in surgery and other disciplines; indeed, the concept of the senior surgical role model is perhaps as old as the discipline itself. Previous authors have demonstrated that mentoring in surgery can have a significant role in career development (5), while others have emphasized the potential benefits in personal and professional development in general (6). Mentoring has been highlighted as a fundamental aspect of training by the Royal College of Surgeons of England and underpins many aspects of its guidance on good surgical practice (7). However, a study of trainees in a single postgraduate school of surgery in the United Kingdom found that the majority did not have a mentor, and a large proportion of those who did were unsatisfied with the quality of mentorship provided (8). Other authors have shown that the implementation of a formal, structured mentor program results in greater satisfaction with the quality of mentorship among trainees compared with an informal, unstructured system (9).

Peer-mentoring has several potential benefits as an adjunct to more traditional models. The mentor and mentee are closer in terms of personal and professional experiences, enabling the peer-mentor to more closely empathize with the challenges faced by the mentee compared to classical mentoring arrangements (10). There is also greater potential for social interaction within the peer-mentor relationship, which in the context of training may increase the sense of community. Peer-mentoring among medical students is well established in many countries (11, 12), and its value in the postgraduate training of healthcare professionals has been described in the fields of academic medicine (13) and nursing (14, 15), among others. Unfortunately, there is currently no system to facilitate peer-mentoring in core surgical training (CST), a 2-year program that constitutes the initial phase of surgical training in the United Kingdom.

## The program

In response to this, a pilot peer-mentoring scheme targeting core surgical trainees within a region of the London postgraduate school of surgery was designed with and approved by the regional training program director, and it was implemented in October 2012 at the start of the academic year. Nine trainees entering the first year of CST (CT1s) were paired (1:1) with a mentor in the second year of CST (CT2s). To facilitate interaction, assignment was based on the geographic location of the initial clinical placement. All trainees were provided with the opportunity to opt-out of the program. Contact details were emailed to both mentors and mentees.

No structured meetings were arranged. Mentors and mentees were advised to contact their assigned ‘partner’ if and when they felt appropriate, and to arrange meetings at intervals of their choosing. Contact details of the trainee responsible for ‘introducing’ the program were also provided to all of the participants as a source of advice should any problems arise.

Preliminary evaluation results suggest that the program was well received. Responses by CT1 mentees (n = 6) and CT2 mentors (n = 6) to an anonymous survey (response rate 67%) revealed that all participants had made contact with their assigned ‘partner,’ and no trainee had opted out of the scheme. Further, no problems with the program implementation were noted.

All CT1 mentees received guidance on at least one aspect of training; 67% (4/6) received guidance on multiple aspects. Support regarding examinations was received by 83% of mentees (5/6), including both the intercollegiate Membership of the Royal College of Surgeons (MRCS) examination and the London School of Surgery's Surgical In-Training Evaluation (SITE). Two-thirds (4/6) of respondents reported receiving guidance on CV development, including how best to prioritize within the first year of CST, and two-thirds received advice on some aspect of the Intercollegiate Surgical Curriculum Program (ISCP) and associated requirements.

All respondents reported that the mentor scheme was a good addition to CST. Comments from trainees regarding adjustments to the scheme included establishing ‘set and protected time for meetings – for example, coinciding with mandatory teaching.’ Another trainee suggested a targeted system to ‘link those who are in need of advice and really keen for a mentor with those who are keen to have a mentee … rather than match on a random basis.’ This was echoed in a comment by another participant that ‘good trainees should seek out their own mentor and mentor others below them.’

## Discussion

To this point, no structured peer-mentoring scheme existed in CST in the United Kingdom. Preliminary results of this pilot effort suggest that a peer-mentoring program is seen as a valuable addition to training by both mentors and mentees – with trainees receiving advice and assistance relating to the specific challenges faced during this phase of surgical training.

In this program, goals were set entirely by the mentee, and all interactions with mentors were organized entirely by participants – with minimal involvement by senior faculty members. However, a number of participants felt the program would benefit from structured meetings at specified intervals, a modification we are considering in future iterations.

Some participants also felt that a scheme targeting trainees in difficulty or those who express a need for mentorship might be more appropriate than simply including all trainees. Indeed, previous studies of peer-mentoring such settings has emphasized the potential of mentoring to relieve stress and anxiety (14, 16), perhaps lending less benefit to trainees who are ‘well adjusted’ to their roles. However, we feel that an opt-out scheme is an appropriate means of involving all trainees who could benefit from peer-mentoring, while avoiding any obligation to participate among those who see little benefit. Whether targeted schemes would be better received by trainees is a topic for further study.

In summary, a peer-mentoring scheme in the early stages of surgical training was easy to introduce and was well received by participating trainees. Furthermore, consideration by postgraduate training institutions should be given to expand such schemes and improve integration of peer-mentorship with other aspects of surgical training. Long-term studies with greater numbers of participants are required to more thoroughly assess the value of peer-mentoring among junior surgical trainees. We plan to further evaluate the value of peer-mentoring in subsequent academic years and involve increasingly larger numbers of participants.
